# Sodium, potassium, and blood pressure regulation in Latin American populations: a critical narrative review of multifactorial determinants

**DOI:** 10.3389/fcvm.2026.1770880

**Published:** 2026-04-10

**Authors:** Ariel Torres, Jose Daniel Sanchez Redroban, Giselle Trujillo, Andres Perez

**Affiliations:** Facultad de Ciencias de la Salud y Bienestar Humano, Universidad Indoamerica, Quito, Ecuador

**Keywords:** cardiovascular prevention, dietary interventions, health equity, hypertension, Latin America, potassium intake, salt sensitivity, social determinants

## Abstract

**Background:**

Salt-sensitive hypertension represents a critical yet inadequately understood public health challenge in Latin America, where sodium intake substantially exceeds international recommendations while potassium consumption remains deficient. The complex interplay between dietary electrolytes, genetic susceptibility, and socioecological determinants necessitates comprehensive analytical frameworks that transcend traditional biomedical models.

**Methods:**

This critical narrative review synthesizes evidence from approximately 154 peer-reviewed publications identified through structured searches in PubMed/MEDLINE, LILACS, SciELO, Scopus, Web of Science, Google Scholar, and SciSpace, covering the period 2015–2025. Sources were selected to represent the breadth of available evidence on sodium and potassium intake patterns, blood pressure regulation, genetic determinants of salt sensitivity, and socioecological contexts in Latin American adult populations. A multi-level analytical framework incorporating physiological mechanisms, epidemiological evidence, genetic determinants, and socioecological contexts was applied for narrative synthesis. Studies published before 2015, including landmark trials and foundational epidemiological work, are cited as background context.

**Results:**

Latin American populations demonstrate consistently elevated sodium excretion (8.4–8.9 g/day salt equivalent) and inadequate potassium intake (1.4–1.5 g/day), yielding unfavorable sodium-to-potassium ratios strongly associated with hypertension prevalence and cardiovascular risk. The landmark Peruvian salt substitution trial demonstrated that community-wide replacement with potassium-enriched alternatives reduced systolic blood pressure by 1.29 mmHg, diastolic by 0.76 mmHg, and incident hypertension by 51% over 30 months. Salt sensitivity exhibits marked heterogeneity, modulated by genetic variants affecting renal sodium handling, obesity, age, and structural determinants governing food access. The sodium-to-potassium ratio emerges as a more robust predictor than either mineral independently.

**Conclusions:**

Understanding hypertension in Latin America requires integrating biological mechanisms with cultural practices, socioeconomic inequalities, and food system transformations. Evidence-based interventions including salt substitution, comprehensive sodium reduction strategies, and potassium enhancement must address implementation barriers including cost, industry engagement, surveillance gaps, and equity dimensions. Future research priorities include standardized exposure assessment protocols, scaled intervention trials, food-source attribution analyses, and socioecological implementation science to translate evidence into sustainable, equitable population health improvements.

## 1 Introduction

Hypertension remains the leading modifiable risk factor for cardiovascular disease globally, with disproportionate burden in low- and middle-income countries including Latin America (1, 2). The relationship between dietary sodium and potassium intake and blood pressure regulation has been established through decades of physiological research and epidemiological investigation (3, 4). However, the translation of this knowledge into effective population-level interventions faces substantial challenges rooted in the complex interplay of biological, behavioral, and structural determinants (5, 6).

Latin America presents a critical case study for examining this multifactorial problem. The region demonstrates consistently elevated sodium consumption averaging 8.4–8.9 g/day salt equivalent—nearly double the World Health Organization recommendation of less than 5 g/day—coupled with inadequate potassium intake of approximately 1.4–1.5 g/day, less than half the recommended 3.5 g/day (7, 8). This unfavorable electrolyte balance occurs within a context of rapid nutrition transition characterized by increasing availability of processed foods, persistent socioeconomic inequalities, diverse genetic backgrounds, and heterogeneous food systems (9, 10).

Recent evidence from Latin America has substantially advanced understanding of sodium-potassium-blood pressure relationships while simultaneously revealing critical knowledge gaps. The landmark stepped-wedge cluster randomized trial conducted in six Peruvian villages demonstrated that community-wide salt substitution with potassium-enriched alternatives (75% sodium chloride, 25% potassium chloride) achieved significant reductions in blood pressure and halved incident hypertension (11). This pragmatic intervention provides proof-of-concept for population-level approaches, yet questions regarding scalability, sustainability, cost-effectiveness, and equity remain inadequately addressed.

Parallel observational research has illuminated important heterogeneity in salt sensitivity across individuals and populations. Studies from Brazil, Mexico, Chile, and other countries document that the sodium-to-potassium ratio may constitute a more informative biomarker than either mineral independently (12–14). Emerging genetic research identifies variants in renal sodium transport pathways that modify blood pressure responses to dietary sodium, with implications for precision nutrition approaches (15, 16). Furthermore, research increasingly recognizes that socioeconomic factors—education, income, food access, cultural practices—fundamentally shape both exposures and health outcomes (17, 18).

Despite this accumulating evidence, critical gaps persist in theoretical frameworks, methodological approaches, and implementation strategies. Most studies invoke established physiological mechanisms without developing explicit socioecological models tailored to Latin American contexts. Methodological heterogeneity in sodium and potassium assessment—ranging from 24-h urine collections to spot urine estimates with varied validation—complicates synthesis and comparability (19, 20). Limited research examines food-level sources of sodium and potassium, hindering targeted reformulation and education strategies. Perhaps most critically, the gap between evidence generation and policy implementation remains substantial, with few studies addressing real-world barriers to translating research findings into sustainable programs.

This critical narrative review was conducted to address these gaps through comprehensive synthesis of the available literature published between 2015 and 2025. The review is organized around three central questions that guided the literature search and synthesis:

1. What are the epidemiological patterns and physiological mechanisms linking sodium, potassium, and blood pressure across diverse Latin American populations, and what genetic and metabolic factors modulate individual salt sensitivity?
2. What socioecological factors shape exposures and outcomes related to dietary electrolytes and hypertension risk in Latin America?
3. What is the effectiveness of available interventions to reduce sodium intake and improve sodium-to-potassium balance in Latin American contexts, and what are the principal implementation challenges?

**Figure 1:**
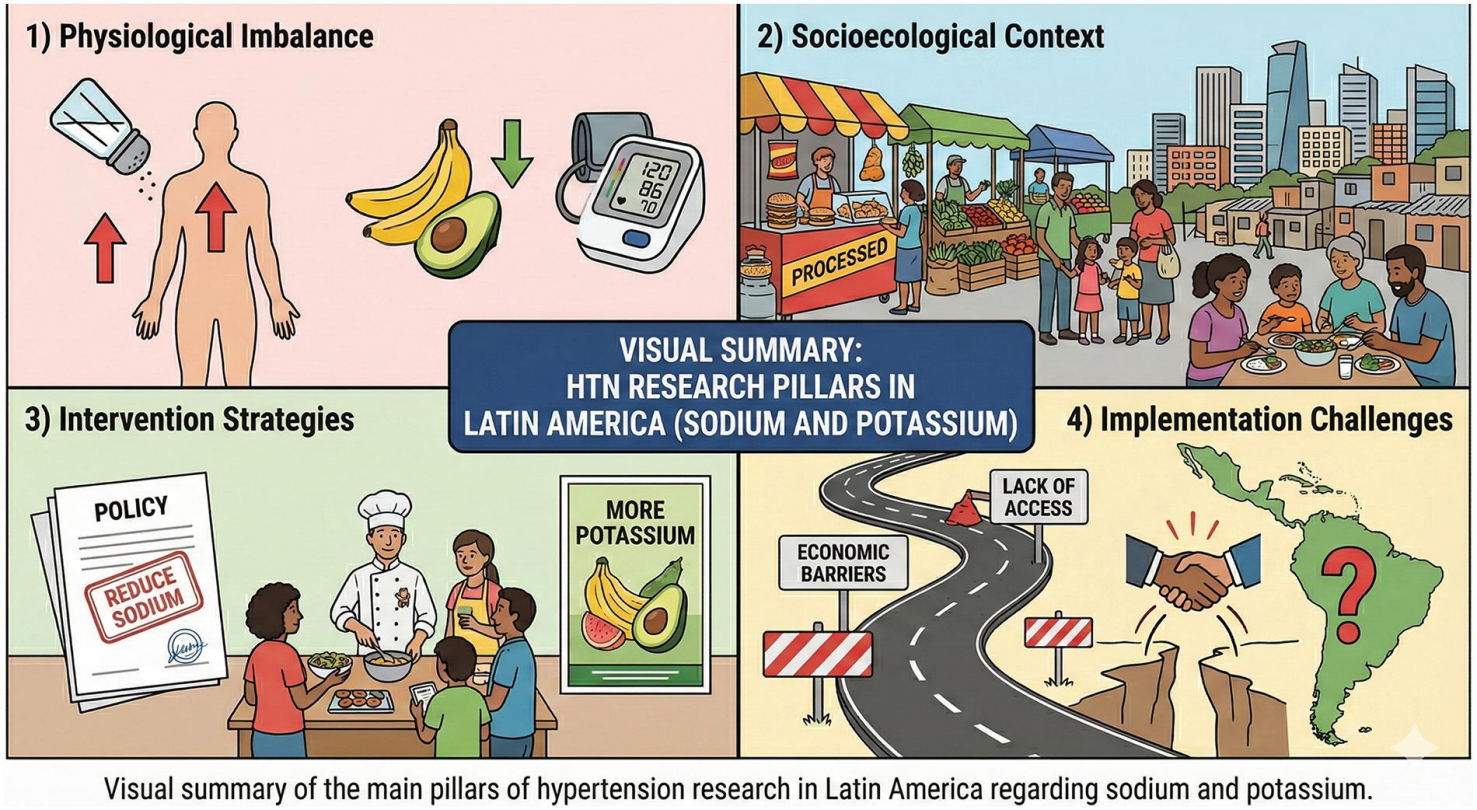
Visual summary of the main pillars of hypertension research in Latin America in relation to sodium and potassium: (1) Physiological imbalance, (2) Socio-ecological context, (3) Intervention strategies, and (4) Implementation challenges.

Our analysis moves beyond simple description to problematize assumptions, identify tensions, and propose pathways toward more contextually appropriate and equitable approaches to hypertension prevention in Latin America (Figure 1).

## 2 Literature search and review approach

### 2.1 Review design

This work constitutes a critical narrative review—a review design that allows for broad, flexible synthesis of a heterogeneous literature, with particular emphasis on theoretical integration, critical appraisal of methodological diversity, and identification of gaps in current knowledge (21, 22). Unlike systematic reviews, narrative reviews do not seek exhaustive enumeration of all available evidence under a pre-specified protocol; instead, they are designed to illuminate complexity, develop conceptual frameworks, and generate hypotheses that more constrained designs may miss. This approach is particularly appropriate for multifactorial public health problems where evidence spans diverse disciplines, study designs, and methodological traditions, as is the case with sodium, potassium, and hypertension in Latin America.

### 2.2 Search strategy

A structured literature search was conducted across seven electronic databases: PubMed/MEDLINE, LILACS (Latin American and Caribbean Health Sciences Literature), SciELO (Scientific Electronic Library Online), Scopus, Web of Science (Core Collection), Google Scholar, and SciSpace. LILACS and SciELO were specifically included to capture peer-reviewed work published in regional journals with limited international indexing, ensuring adequate representation of Latin American research. Searches were conducted in English, Spanish, and Portuguese.

Search terms combined concepts related to sodium and potassium intake (e.g., *sodium intake, salt intake, dietary sodium, potassium intake, sodium-to-potassium ratio*), blood pressure and cardiovascular outcomes (e.g., *hypertension, blood pressure, cardiovascular disease, salt sensitivity*), and Latin American geographic scope (e.g., *Latin America, South America, Brazil, Mexico, Chile, Colombia, Argentina, Peru, Ecuador, Hispanic, Latino*). The search strategy was adapted for each database, incorporating controlled vocabulary (e.g., MeSH terms in PubMed, DeCS in LILACS) and field tags where applicable. The complete search strings for all databases, including any filters applied (e.g., language, publication date), are provided in the Supplementary Table S1.

Supplementary searches addressed specific subtopics including salt substitution, food labeling, food reformulation, genetic determinants of salt sensitivity, and socioeconomic determinants of dietary patterns. The reference lists of key included studies and relevant systematic reviews were also hand-searched to identify additional potentially eligible publications.

The search was primarily oriented toward studies published between January 2015 and December 2025. This decade-long window was chosen to capture the period following major national sodium reduction policy initiatives in Chile, Brazil, and Argentina, and reflects the widespread adoption of urinary biomarker methods for population-level dietary sodium assessment, ensuring the synthesized evidence is relevant to current policy contexts. Earlier landmark studies—including foundational epidemiological work (e.g., INTERSALT) and pivotal randomized trials—are incorporated in the Introduction and Discussion as necessary background and context.

### 2.3 Study selection and eligibility criteria

Studies were considered eligible for inclusion if they: (1) were peer-reviewed original research or systematic reviews; (2) included adult populations (18 years); (3) were conducted in Latin American countries or in U.S. Hispanic populations with clear relevance to the region; (4) reported on at least one of the following: sodium or potassium intake/excretion, the sodium-to-potassium ratio, blood pressure or hypertension outcomes, salt sensitivity, or related interventions; and (5) were published in English, Spanish, or Portuguese. No restrictions were placed on study design to capture the breadth of evidence from observational studies to randomized trials.

Studies were excluded if they focused exclusively on pediatric populations, were conference abstracts or editorials without original data, or did not provide sufficient quantitative data on exposures or outcomes of interest. The study selection process was conducted independently by two reviewers (A.T. and A.P.). Titles and abstracts were screened against the eligibility criteria, and the full texts of potentially relevant articles were retrieved and assessed. Disagreements were resolved through consensus or consultation with a third reviewer (J.S.). A PRISMA-style flow diagram detailing the selection process is available in the Supplementary Figure S1.

### 2.4 Source selection and synthesis

Sources were selected to represent the breadth and diversity of available evidence across study designs, geographic coverage, and thematic domains. Approximately 154 peer-reviewed publications were identified and reviewed, spanning the disciplines of epidemiology, cardiology, nutrition science, human genetics, health economics, and implementation science.

Given the heterogeneity of study designs and analytical approaches represented in the reviewed literature, a quantitative pooling of results was not attempted. Instead, evidence is synthesized narratively, organized by the three guiding questions articulated in the Introduction. Throughout the text, the strength and limitations of available evidence are discussed explicitly, with attention to study design hierarchy, exposure measurement quality, sample representativeness, and the degree to which findings can support causal inference. Table 1 provides a descriptive overview of the reviewed literature by study design category.

**Table 1:** Overview of reviewed literature by study design category (approximately N=154 publications).

Design	n (approx. %)	Primary countries	Main topics
Systematic reviews/meta-analyses	18 (11.7)	Multi-country	Na/K intake, BP, CV events
Randomized/stepped-wedge trials	9 (5.8)	Peru, Brazil, Chile	BP, incident HTN
Prospective cohort studies	22 (14.3)	Brazil, Mexico, Chile	BP change, CV mortality
Cross-sectional/national surveys	62 (40.3)	Mexico, Ecuador, Uruguay, Colombia, Dominican Republic, Chile	Na/K excretion, HTN prevalence
Genetic/mechanistic studies	15 (9.7)	Brazil, Colombia, Argentina	Salt sensitivity, genetic variants
Policy/implementation studies	18 (11.7)	Chile, Brazil, Argentina, Peru	Intervention effectiveness
Economic evaluations	10 (6.5)	Brazil, Chile, Multi-country	Cost-effectiveness, DALYs
Total	154 (100)		

BP, blood pressure; CV, cardiovascular; DALYs, disability-adjusted life years; HTN, hypertension; Na/K, sodium-to-potassium ratio.

### 2.5 Quality appraisal and certainty of evidence

As this is a narrative review encompassing a wide range of study designs (from qualitative research to randomized trials), a formal risk of bias assessment using a single tool was not performed. However, the methodological quality of the included studies was critically considered throughout the synthesis. For key bodies of evidence, such as the observational studies on sodium intake and the randomized trials of salt substitution, we have explicitly commented on potential biases (e.g., selection bias, measurement error, confounding) and their implications for the interpretation of findings. This critical appraisal is integrated into the narrative for each thematic section, allowing for a nuanced discussion of the strength of the evidence base.

## 3 Theoretical and conceptual frameworks

### 3.1 Physiological mechanisms of blood pressure regulation

The fundamental relationship between dietary sodium, potassium, and blood pressure operates through well-established yet incompletely understood physiological mechanisms. Excessive sodium intake promotes fluid retention through osmotic pressure changes and increases peripheral vascular resistance through direct effects on vascular smooth muscle and endothelial function (16, 23). These effects are mediated by the renin-angiotensin-aldosterone system (RAAS), which responds to changes in extracellular sodium concentration to regulate blood volume and vascular tone. Potassium exerts countervailing effects through multiple pathways. It facilitates renal sodium excretion (natriuresis) by inhibiting sodium reabsorption in the distal tubule, promotes vasodilation through effects on endothelial nitric oxide production and vascular smooth muscle hyperpolarization, and modulates sympathetic nervous system activity (24, 25). The sodium-to-potassium ratio thus captures the net effect of these opposing forces, potentially explaining why it emerges as a stronger predictor of blood pressure changes than either mineral independently (13, 26).

However, physiological mechanisms alone cannot account for the substantial heterogeneity observed across individuals and populations. Salt sensitivity—defined as blood pressure changes exceeding 5–10 mmHg in response to acute sodium loading or restriction—affects an estimated 25%–50% of hypertensive and 15%–25% of normotensive individuals, with marked variation by age, ethnicity, body composition, and comorbidities (15). This heterogeneity reflects complex interactions between genetic variants, kidney function, hormonal regulation, inflammatory processes, and metabolic factors that remain incompletely characterized, particularly in Latin American populations.

### 3.2 Genetic determinants and precision nutrition

Emerging research has identified multiple genetic loci associated with salt-sensitive hypertension, primarily involving genes regulating renal sodium handling. Variants in genes encoding components of the epithelial sodium channel (ENaC), sodium-chloride cotransporter (NCC), and other renal tubular transport proteins influence the kidney’s capacity to excrete sodium loads (27, 28). Polymorphisms in the angiotensinogen gene (AGT) and angiotensin-converting enzyme (ACE) modify RAAS activity, altering blood pressure responses to sodium intake (29). Recent genome-wide association studies in Brazilian populations have identified novel loci associated with blood pressure and hypertension in individuals of African ancestry, highlighting the importance of conducting genetic research in diverse populations rather than extrapolating from studies in populations of European descent (30).

These findings suggest that precision nutrition approaches tailored to genetic profiles may eventually enhance intervention effectiveness, though substantial research is needed to validate genetic markers, establish clinical utility, and ensure equitable access to personalized approaches. Beyond genetics, epigenetic modifications—changes in gene expression without alterations in DNA sequence—may mediate long-term effects of early-life sodium exposure on blood pressure regulation. Limited research in Latin American populations examines these mechanisms, representing an important frontier for understanding developmental origins of hypertension and potential intergenerational effects.

### 3.3 Socioecological framework: the missing dimension

A critical gap in the literature is the absence of explicit, comprehensive socioecological frameworks developed specifically for understanding sodium-potassium-blood pressure relationships in Latin American contexts. While individual studies acknowledge social determinants, the field lacks published models that systematically map multilevel determinants and intervention points.

An adequate socioecological framework for this problem must integrate at least five levels:

**Individual level:** Biological susceptibility (genetics, kidney function, metabolic factors), health literacy, dietary preferences, and self-management behaviors.
**Interpersonal level:** Family food preparation practices, household purchasing decisions, cultural food traditions, and social support for dietary modification.
**Organizational level:** Availability and pricing of low-sodium and potassium-rich foods in food retail environments, workplace and school food policies, and healthcare system capacity for hypertension screening and management.
**Community level:** Food environment characteristics (density of grocery stores vs. fast food outlets, availability of fresh produce), community norms regarding salt use and dietary practices, and social cohesion facilitating collective action.
**Policy level:** National sodium reduction strategies, food industry reformulation requirements or voluntary agreements, front-of-package labeling regulations, agricultural subsidies affecting food prices, and trade policies influencing food imports.

The interaction across these levels determines both population exposure patterns and the feasibility and effectiveness of interventions. For example, individual-level nutrition education campaigns may fail if the local food environment offers limited access to affordable, low-sodium alternatives—a structural constraint requiring policy-level intervention. Similarly, industry reformulation may achieve population-wide sodium reduction more efficiently than individual behavior change approaches, yet faces implementation barriers related to taste preferences, technological feasibility, and economic incentives requiring multi-stakeholder engagement.

Developing and testing explicit socioecological frameworks represents a critical priority for advancing both research and practice in Latin America.

## 4 Methodological approaches and measurement considerations

This section reviews methodological issues pertaining to the primary studies included in this review—specifically, challenges in measuring sodium and potassium intake—rather than the review approach itself (described in Section 2 above).

### 4.1 Sodium and potassium assessment methods

#### 4.1.1 24-h urinary excretion

Complete 24-h urine collection is considered the gold standard for population-level assessment, as it captures intake from all sources (added salt, processed foods, natural food content) and reflects actual consumption rather than reported intake (19, 20). Under steady-state conditions, approximately 90% of dietary sodium and 70%–90% of potassium is excreted in urine, enabling reasonably accurate estimation of intake. However, 24-h collection faces practical challenges including participant burden, incomplete collection (potentially introducing selection bias if completeness correlates with both exposure and outcome), requirements for specimen storage and transport, and higher cost compared to other methods. Studies in Mexico and Uruguay utilizing 24-h collections report completion rates of 60%–85%, with higher exclusion rates in population-based surveys compared to clinical research settings (19, 20).

#### 4.1.2 Spot urine with estimation equations

To enhance feasibility for large-scale surveillance, national surveys increasingly employ single spot urine samples combined with prediction equations (e.g., Kawasaki, Tanaka, INTERSALT formulae) to estimate 24-h excretion (14). These methods dramatically reduce participant burden and study costs, enabling inclusion in multipurpose health surveys. Critical limitations include substantial individual-level measurement error, as spot samples reflect point-in-time concentration affected by recent intake, hydration status, and time of day. While population mean estimates may be unbiased if sampling is random with respect to timing, individual-level misclassification attenuates associations between exposures and outcomes, potentially underestimating true effect sizes (31). Furthermore, estimation equations developed in specific populations may not perform equivalently across different ethnic groups, dietary patterns, and body compositions, raising questions about generalizability to diverse Latin American settings (14). Recent validation studies comparing spot estimates to gold-standard 24-h collections in Latin American populations report variable agreement, with some equations substantially over- or underestimating intake in specific subgroups (32).

#### 4.1.3 Dietary assessment methods

Self-reported dietary intake via 24-h recalls or food frequency questionnaires enables examination of food sources and dietary patterns but suffers from well-documented limitations including recall bias, social desirability bias, and difficulty accurately estimating sodium content given high variability in processing and preparation (33). Potassium intake may be somewhat more accurately assessed, as it primarily derives from recognizable whole foods (fruits, vegetables, legumes) rather than ubiquitous “hidden” sources. Studies linking dietary recalls with urinary biomarkers in Latin American populations are surprisingly rare, representing a critical gap for informing food-based intervention strategies.

### 4.2 Study design considerations

#### 4.2.1 Observational studies

Cross-sectional analyses using national health survey data have provided valuable population-level prevalence estimates and exposure-outcome associations across multiple Latin American countries (12, 14). However, cross-sectional designs cannot establish temporal ordering or rule out reverse causation, limiting causal inference. Prospective cohort studies, particularly ELSA-Brasil with its repeated measures design, enable stronger causal inference by examining within-person changes in sodium-to-potassium ratio and blood pressure over time (13). Multi-country comparative studies provide insights into regional heterogeneity and potential moderating effects of population characteristics and food systems (34). However, differences in assessment methods, adjustment for covariates, and outcome definitions complicate direct comparison and pooled analysis across studies.

#### 4.2.2 Intervention studies

The Peruvian salt substitution trial represents a landmark contribution, employing a stepped-wedge cluster randomized design that provides strong causal evidence while maintaining pragmatic implementation in real-world community settings (11). The cluster randomization (by village) and stepped implementation (with sequential rollout across clusters) enabled evaluation of population-level effects while accounting for temporal trends and minimizing contamination. Several methodological features enhance the trial’s validity and applicability. First, the intervention targeted household salt replacement rather than individual behavior change, potentially achieving greater reach and equity than education-based approaches. Second, intention-to-treat analysis preserving cluster assignment provides conservative estimates of effectiveness under real-world conditions of imperfect adherence. Third, the trial measured both blood pressure changes and incident hypertension, demonstrating benefits for prevention as well as treatment.

### 4.3 Analytical approaches

Statistical methods employed across studies reflect increasing sophistication but also introduce heterogeneity that complicates synthesis. Most studies employ multivariable regression adjusting for age, sex, body mass index, and sociodemographic factors, though specific covariate sets vary. Handling of antihypertensive medication differs substantially, with some studies excluding treated individuals, others adjusting for treatment, and still others adding constant values to observed blood pressure for treated individuals—approaches that can yield different conclusions about exposure-outcome relationships (35). Stratified analyses and tests for effect modification enable examination of heterogeneous treatment effects across subgroups defined by hypertension status, age, sex, obesity, and genetic variants.

## 5 Epidemiological patterns and key findings

### 5.1 Overview of reviewed literature

The reviewed literature spans diverse designs, countries, and outcomes, as summarized in Table 1. The evidence base is dominated by cross-sectional studies using nationally representative survey data, limiting causal inference at the population level. Prospective cohort evidence is largely anchored by the Brazilian ELSA-Brasil cohort and the Hispanic Community Health Study/Study of Latinos (HCHS/SOL) for U.S. Hispanic populations. Interventional evidence from the region is limited but of high methodological quality, anchored by the Peruvian stepped-wedge trial.

### 5.2 Sodium intake levels and trends

Population-level surveillance across Latin America consistently documents sodium intake substantially exceeding WHO recommendations. Studies employing 24-h urinary collection or validated spot urine methods report mean sodium excretion of 3,200–3,500 mg/day (equivalent to 8–9 g/day salt), with wide variation across individuals and settings (7, 8). Mexican national survey data from ENSANUT reveal particularly high sodium intake with mean excretion of 3,354 mg/day overall, and even higher levels among men (3,700 mg/day) compared to women (3,000 mg/day) (14). Similar patterns emerge in Uruguay (mean 3,560 mg/day), Chile (3,200 mg/day), and Dominican Republic (3,400 mg/day) (12, 20, 36). Notably, these estimates are generally conservative, as they may not fully capture sodium from all sources and exclude individuals with incomplete collections.

Age patterns show relatively stable or slightly increasing sodium intake across adulthood, though some studies report higher intake in middle-aged adults with modest declines in elderly populations potentially reflecting reduced food consumption or survivor bias (37). Socioeconomic gradients in sodium intake appear complex and context-specific, with some settings showing higher intake in lower socioeconomic groups (potentially reflecting greater reliance on processed foods) while others show the opposite pattern (10). Limited longitudinal data on temporal trends in sodium intake hinders assessment of whether recent policy initiatives and voluntary industry reformulation efforts have achieved meaningful population-level reductions. Establishing routine, high-quality surveillance systems represents a critical priority for monitoring progress and holding stakeholders accountable.

### 5.3 Potassium intake deficiency

In stark contrast to excessive sodium intake, Latin American populations demonstrate consistently inadequate potassium consumption. Population studies report mean potassium excretion of approximately 1,400–1,500 mg/day, less than half the WHO recommendation of 3,510 mg/day (8, 14, 36). This profound potassium deficit reflects low consumption of potassium-rich foods including fruits, vegetables, legumes, and dairy products. Small-scale dietary studies document particularly low fruit and vegetable intake among elderly and lower-income populations, with average servings well below recommended levels (18). These patterns reflect multiple barriers including cost (fresh produce is often more expensive than processed foods), availability (limited access to markets with diverse fresh produce in some settings), storage (lack of refrigeration), preparation time and skills, and taste preferences shaped by lifelong dietary patterns. The public health implications of concurrent sodium excess and potassium deficiency are substantial, creating maximally unfavorable conditions for blood pressure regulation.

### 5.4 Sodium-to-potassium ratio: a superior biomarker

Emerging evidence strongly suggests that the urinary sodium-to-potassium (Na/K) ratio constitutes a more informative biomarker for blood pressure and cardiovascular risk than either mineral independently (8, 12, 13). The longitudinal ELSA-Brasil study examining within-person changes over four years documented stronger associations between changes in Na/K ratio and blood pressure changes compared to sodium or potassium alone (13). Participants in the highest quintile of Na/K ratio increase experienced substantially greater blood pressure elevations compared to those with stable or decreasing ratios, even after adjustment for multiple confounders including body mass index, diet quality, physical activity, and medication use.

Cross-sectional analyses in Chilean national survey data similarly identified Na/K ratio as the strongest predictor of blood pressure categories among electrolyte measures examined (12). Individuals in the highest Na/K ratio quartile had 2.4-fold increased odds of hypertension compared to the lowest quartile, after multivariable adjustment. These findings suggest that public health strategies should emphasize improving the Na/K ratio through simultaneous sodium reduction and potassium enhancement, rather than focusing exclusively on sodium restriction.

### 5.5 Blood pressure associations and cardiovascular outcomes

Studies consistently document positive associations between sodium intake and blood pressure across Latin American populations, with evidence of dose-response relationships (5, 37). Meta-analyses of Latin American studies estimate that each 1,000 mg/day increase in sodium intake is associated with approximately 1–2 mmHg higher systolic blood pressure and 0.5–1 mmHg higher diastolic blood pressure, with somewhat larger effects in hypertensive compared to normotensive individuals (7). While these effect sizes may appear modest, their population-level implications are substantial. Even small mean blood pressure reductions translate to meaningful reductions in cardiovascular events at the population level, as risk operates continuously across the blood pressure distribution (38).

Prospective cohort studies linking sodium intake to cardiovascular outcomes have yielded more heterogeneous results, with some showing positive associations particularly at very high sodium intakes while others report U-shaped or J-shaped relationships (6, 34). These inconsistencies have generated substantial scientific debate regarding optimal sodium intake levels and whether aggressive restriction below 2,000 mg/day confers benefits or potential harms (39, 40). Despite these controversies, international guideline bodies including WHO, American Heart Association, and European Society of Cardiology/Hypertension continue to recommend population-level sodium reduction as a cost-effective strategy for hypertension prevention (2, 41, 42).

## 6 Intervention evidence and implementation challenges

### 6.1 The peruvian salt substitution trial: a landmark study

The stepped-wedge cluster randomized trial conducted by Bernabe-Ortiz et al. (11) in six villages in Tumbes, Peru, represents the strongest evidence to date for a scalable population-based intervention to reduce blood pressure and prevent hypertension in Latin America. The intervention involved household-level replacement of regular salt with a potassium-enriched substitute (approximately 75% sodium chloride, 25% potassium chloride) provided free of charge to all village residents. Key findings over 30 months of follow-up included: (1) significant reduction in mean systolic blood pressure of 1.29 mmHg (95% CI: 0.55–2.03) and diastolic blood pressure of 0.76 mmHg (95% CI: 0.24–1.28); (2) 51% reduction in incident hypertension among individuals without hypertension at baseline (HR 0.49, 95% CI: 0.31–0.78); (3) larger absolute blood pressure reductions among individuals with baseline hypertension; and (4) significant increases in urinary potassium excretion and decreases in Na/K ratio.

Several design features enhance the trial’s validity and interpretability. The stepped-wedge design enabled all communities to receive the intervention while providing rigorous comparison across implementation periods. Cluster randomization by village minimized contamination while reflecting real-world implementation at community level. Intention-to-treat analysis preserving cluster assignment provided conservative estimates of effectiveness. Importantly, the intervention targeted household salt replacement rather than individual behavior change, potentially achieving greater reach, equity, and sustainability than education-based approaches.

### 6.2 Remaining questions and implementation challenges

Despite the trial’s methodological rigor and encouraging findings, substantial questions regarding scalability, sustainability, cost-effectiveness, and equity remain inadequately addressed.

#### 6.2.1 Scalability and delivery models

The trial provided salt substitutes free of charge with intensive community engagement in rural villages—conditions that differ substantially from routine programmatic implementation at regional or national scale. Key questions include what delivery models (free distribution, subsidized sales, market-based approaches) achieve adequate population coverage and equity; how effectiveness and uptake vary across urban vs. rural settings; what community engagement and education strategies are necessary to ensure acceptance and sustained use; and how supply chains can be established to ensure consistent availability of salt substitutes. Pilot programs testing alternative delivery models in diverse settings would provide critical evidence for policy decisions.

#### 6.2.2 Cost-effectiveness

While salt substitutes incur modest incremental cost compared to regular salt (estimated at $1–2 USD annually per capita), comprehensive economic evaluation requires consideration of multiple factors including production and distribution costs, healthcare savings from prevented cardiovascular events, quality-adjusted life years gained, and budget impact for different financing mechanisms. Modeling studies suggest favorable cost-effectiveness ratios, with some estimates indicating cost savings within 10–15 years (43). However, these analyses employ assumptions regarding effectiveness, coverage, sustained use, and healthcare costs that require empirical validation.

#### 6.2.3 Safety considerations

Potassium-enriched salt substitutes are generally safe for populations with normal kidney function, as healthy kidneys readily excrete excess potassium. However, individuals with advanced chronic kidney disease or those taking certain medications (potassium-sparing diuretics, ACE inhibitors, angiotensin receptor blockers) may be at risk for hyperkalemia if consuming large amounts of potassium chloride. Population-based implementation therefore requires careful consideration of safety monitoring, contraindications, and mechanisms to identify and counsel higher-risk individuals. The Peruvian trial excluded individuals with estimated glomerular filtration rate below 45 mL/min/1.73 m^2^ and detected no serious adverse events related to hyperkalemia (11).

#### 6.2.4 Industry engagement and food system transformation

Salt substitution targeting household discretionary salt represents only one component of comprehensive sodium reduction strategies. In many Latin American countries, processed and industrialized foods contribute 30%–50% or more of total sodium intake (43). Effective population-level sodium reduction therefore requires engagement with food manufacturers to reformulate products. Several policy approaches exist along a continuum from voluntary to mandatory: (1) voluntary reformulation agreements with industry; (2) mandatory reformulation targets with staged implementation timelines (Chile, Argentina models); (3) taxation of high-sodium products; and (4) procurement standards for institutional food service. Chile’s comprehensive Law of Food Labeling and Advertising, implemented in 2016, includes sodium thresholds triggering front-of-package warning labels and restrictions on marketing to children (44).

### 6.3 Other intervention approaches

Beyond salt substitution and food reformulation, evidence from Latin America regarding other intervention modalities remains limited. Consumer education and mass media campaigns can reach large audiences but evidence regarding effectiveness for sustained sodium reduction is mixed, with most studies showing modest short-term changes that often decay over time (45). Community-based participatory interventions in Peru and elsewhere have implemented gradual salt reduction in community kitchens, demonstrating feasibility and some blood pressure benefits (46). Healthcare system interventions strengthening hypertension screening and treatment in primary care settings represent essential complementary strategies, though these do not address primary prevention (47).

## 7 Salt sensitivity: heterogeneity and determinants

### 7.1 Genetic determinants

Salt sensitivity—the phenomenon whereby blood pressure responds substantially to changes in sodium intake—exhibits marked interindividual variability influenced by genetic, metabolic, and environmental factors. Genetic research has identified multiple variants associated with salt sensitivity, primarily involving genes regulating renal sodium handling (15, 27). The epithelial sodium channel (ENaC), expressed in the distal nephron, plays a central role in fine-tuning sodium reabsorption. Studies in Brazilian populations have documented associations between genetic variants and blood pressure responses to sodium intake (28, 30). A genome-wide association study in African-Brazilian quilombo communities identified novel loci associated with hypertension, emphasizing the importance of conducting genetic research in diverse populations (30). Polymorphisms in the renin-angiotensin-aldosterone system genes also influence salt sensitivity through effects on sodium retention and vascular function (29).

### 7.2 Metabolic and physiological modulators

Beyond genetics, multiple metabolic and physiological factors modulate salt sensitivity. Even mild reductions in glomerular filtration rate impair the kidney’s capacity to excrete sodium loads, increasing salt sensitivity (16). Chronic kidney disease prevalence is substantial in Latin America, driven by diabetes, hypertension, and potentially environmental exposures (48). Obesity enhances salt sensitivity through multiple mechanisms including increased renal sodium reabsorption, sympathetic nervous system activation, and inflammatory processes (23). The high prevalence of obesity across Latin America (20%–30% in most countries) suggests that a substantial proportion of the population may exhibit enhanced salt sensitivity. Salt sensitivity also increases with aging, potentially reflecting age-related declines in kidney function and vascular stiffness (49).

### 7.3 Implications for intervention design

Heterogeneity in salt sensitivity raises important questions for intervention design and equity. Universal approaches may achieve greater population impact and avoid stigmatization, but targeted approaches may be more cost-effective if concentrated among most responsive individuals. The Peruvian trial’s finding that both hypertensive and normotensive individuals benefited suggests value in universal approaches that provide benefits across risk strata (11). If salt sensitivity correlates with socioeconomic position (through associations with obesity, kidney disease, or comorbidities), population-level sodium reduction may yield greater absolute benefits for disadvantaged groups—a “proportionate universalism” that reduces disparities (50).

## 8 Socioecological determinants and structural barriers

### 8.1 Food environment and processed foods

The nutrition transition in Latin America has been characterized by rapid increases in availability, affordability, and consumption of processed and industrially manufactured foods—products typically high in sodium, added sugars, unhealthy fats, and additives while low in fiber, vitamins, and minerals including potassium (51). Studies across the region document that such processed foods contribute 20%–40% or more of total energy intake, with higher proportions among urban, younger, and higher-income populations (52). The sodium density of processed foods substantially exceeds that of minimally processed foods, while potassium content is generally lower, creating a doubly unfavorable nutritional profile.

The food environment—the physical, economic, and sociocultural context shaping dietary behaviors—powerfully influences sodium and potassium intake patterns:

**Availability:** Urban food environments often feature high density of fast-food restaurants, convenience stores, and supermarkets offering abundant processed options, while fresh produce markets may be less accessible, particularly in lower-income neighborhoods (53).
**Affordability:** Economic analyses consistently show that processed foods provide calories at lower cost than fresh fruits, vegetables, and other minimally processed foods—a price differential that disproportionately affects lower-income households (54).
**Marketing and advertising:** Aggressive promotion of processed foods, particularly to children and adolescents, shapes preferences, brand loyalty, and consumption patterns (55).
**Convenience:** Processed foods require minimal preparation time and skills compared to cooking from fresh ingredients—an important consideration for households with time poverty.

Addressing these structural determinants requires comprehensive policy interventions operating at multiple levels: agricultural policies affecting relative prices of healthy vs. unhealthy foods; urban planning ensuring equitable access to food retail; marketing restrictions protecting children from commercial exploitation; and social policies addressing time and economic poverty constraining healthy dietary behaviors.

### 8.2 Cultural and social practices

Dietary patterns are deeply embedded in cultural traditions, family practices, and social identities. Qualitative research in Latin American communities reveals complex attitudes toward salt reduction. Some individuals express skepticism that taste can be maintained with less salt, concern that family members will reject low-salt food, or belief that salt reduction is unnecessary for those without diagnosed hypertension (56). Interventions that fail to engage with these cultural dimensions risk low acceptability and adherence. Promising approaches include participatory recipe adaptation working with community members to gradually reduce salt while maintaining acceptable taste; positive framing emphasizing addition of potassium-rich foods rather than solely focusing on sodium restriction; social norm change leveraging peer influence; and culinary skills training emphasizing flavor development through techniques other than high sodium addition (46).

### 8.3 Socioeconomic position and health inequities

Substantial evidence documents socioeconomic gradients in hypertension prevalence and control, with lower education and income associated with higher blood pressure, lower awareness and treatment rates, and worse outcomes (18, 34). These disparities reflect multiple intersecting pathways:

**Differential exposure:** Lower socioeconomic groups may have higher sodium intake or lower potassium intake due to greater reliance on inexpensive processed foods and limited access to fresh produce.
**Differential susceptibility:** Higher prevalence of obesity, diabetes, chronic kidney disease, and chronic stress among disadvantaged populations may increase salt sensitivity and hypertension risk.
**Differential healthcare access:** Economic and geographic barriers to healthcare, lower health literacy, and experiences of discrimination within healthcare settings impede hypertension screening, diagnosis, and treatment among disadvantaged groups.
**Differential impacts of interventions:** Interventions implemented without attention to equity may preferentially benefit advantaged groups, thereby widening disparities even as population means improve.

Achieving equitable reductions in hypertension requires that interventions explicitly address these disparities through: ensuring affordability and accessibility of low-sodium, potassium-rich foods through subsidies, social protection programs, or price regulations; targeting interventions to high-risk communities and populations; strengthening primary healthcare capacity in underserved areas; engaging community health workers and peer educators; and monitoring and evaluating equity impacts using socioeconomic stratification.

## 9 Research gaps and future directions

### 9.1 Measurement and surveillance

Harmonized, validated methods for sodium and potassium assessment across Latin American countries would enable pooled analyses, trend monitoring, and cross-country comparisons. Priorities include: periodic nationally representative surveys using 24-h urine collections with standardized quality control; validation of spot urine estimation equations in diverse Latin American populations; and linkage of urinary biomarkers with detailed dietary intake data to quantify food sources. Integrating sodium and potassium biomarkers into routine population health surveys would enable monitoring of trends and evaluation of policy impacts.

### 9.2 Intervention research

Replication and adaptation of the Peruvian salt substitution model in diverse urban and rural settings across multiple countries, testing different delivery models, target populations, and implementation strategies, is a research priority. Rigorous evaluation of comprehensive sodium reduction strategies including mandatory reformulation, taxation, marketing restrictions, and procurement standards should complement household-level interventions. Natural experiment designs exploiting policy variation across jurisdictions can provide strong quasi-experimental evidence when randomized trials are infeasible. Explicitly examining how interventions affect different socioeconomic groups is essential for identifying barriers and facilitators to equitable reach and effectiveness.

### 9.3 Mechanistic and translational research

Integrating genomics, metabolomics, and microbiome analyses into cohort studies and trials would identify biomarkers of salt sensitivity, understand gene-environment interactions, and explore potential for precision nutrition approaches. Expanded cohort studies with extended follow-up linking sodium and potassium exposures to hard cardiovascular endpoints are needed, with adequate power to examine effect modification by baseline blood pressure, age, sex, ethnicity, and comorbidities. Investigating effects of maternal and early-life sodium exposure on blood pressure trajectories may inform interventions targeting pregnancy and early childhood.

### 9.4 Food sources and dietary patterns

Comprehensive dietary assessment studies combining 24-h recalls or food records with urinary biomarkers to quantify contributions of specific foods to total sodium and potassium intake are essential for prioritizing reformulation targets. Examining sodium and potassium in context of overall dietary patterns, processing levels, and dietary diversity—comparing associations of isolated nutrients vs. dietary pattern scores adapted to Latin American contexts—represents an important research frontier.

### 9.5 Economic evaluation and socioecological models

Comprehensive economic evaluations of different intervention strategies using region-specific cost and epidemiological data, including distributional analyses examining equity implications, are needed to inform policy priorities. Developing and testing explicit socioecological models that map multilevel determinants of sodium and potassium intake and blood pressure in Latin American contexts, and applying established implementation science frameworks (e.g., RE-AIM, CFIR) to generate generalizable knowledge about what works, for whom, in what contexts, and why, represent important methodological priorities.

## 10 Policy recommendations

Based on synthesis of available evidence, we propose the following policy recommendations for reducing hypertension burden in Latin America:

### 10.1 Priority interventions

1. **Scale salt substitution programs:** Building on proven effectiveness in Peru, governments should pilot salt substitution programs in diverse settings, testing delivery models including subsidized distribution to high-risk populations, market-based approaches with consumer education, or mandatory reformulation of packaged salt products. Programs should include rigorous monitoring of coverage, safety, effectiveness, and equity impacts.
2. **Comprehensive sodium reduction strategies:** Implement multi-component approaches including:
  - Mandatory or voluntary sodium reduction targets for processed foods with staged timelines
  - Front-of-package warning labels for high-sodium products
  - Restrictions on marketing of high-sodium products to children
  - Procurement standards for institutional food service (schools, hospitals, government facilities)
  - Consumer education campaigns integrated with environmental changes
3. **Promote potassium-rich diets:** Increase availability and affordability of fruits, vegetables, legumes, and other potassium-rich foods through agricultural subsidies, market interventions, school and workplace feeding programs, and nutrition education emphasizing positive addition of healthy foods rather than solely restriction of unhealthy foods.
4. **Prioritize sodium-to-potassium ratio:** Surveillance systems should routinely measure both sodium and potassium, report Na/K ratios, and evaluate interventions based on improvements in this integrated biomarker rather than sodium reduction alone.

### 10.2 Strengthening infrastructure

5. **Establish surveillance systems:** Invest in periodic nationally representative surveys using standardized 24-h urine collection protocols, validate spot urine methods in local populations, and link dietary intake data with biomarkers to inform policy decisions.
6. **Build healthcare capacity:** Strengthen primary care systems for hypertension screening, diagnosis, treatment, and follow-up, with particular attention to underserved populations and communities. Implement task-shifting, team-based care, and community health worker programs to improve access and coverage.

### 10.3 Addressing equity

7. **Equity-focused implementation:** Design interventions with explicit attention to reaching and benefiting disadvantaged populations. Consider targeted approaches for low-income communities, subsidies ensuring affordability of healthy options, and community engagement strategies addressing cultural and linguistic diversity.
8. **Monitor equity impacts:** Routinely stratify surveillance and evaluation data by socioeconomic position, geography, ethnicity, and other equity dimensions. Use findings to identify disparities and adapt interventions to reduce rather than exacerbate inequities.

### 10.4 Multi-sectoral coordination

9. **Whole-of-government approaches:** Coordination across health, agriculture, trade, education, and social protection sectors to create policy coherence supporting healthy dietary behaviors. Engage stakeholders including industry, civil society, academia, and communities in multi-stakeholder platforms addressing conflicts of interest and power imbalances.
10. **Regional collaboration:** Strengthen regional cooperation through Pan American Health Organization and other mechanisms to share best practices, harmonize methods, coordinate research, and develop regional policy frameworks while allowing country-specific adaptation.

## 11 Discussion

This critical narrative review synthesizes evidence from approximately 154 publications examining sodium, potassium, and blood pressure relationships in Latin American populations over the past decade. The following discussion addresses the three guiding questions stated in the Introduction.

### 11.1 Epidemiological patterns and physiological mechanisms

Evidence is robust regarding consistently elevated sodium intake and deficient potassium intake across Latin American populations, yielding unfavorable Na/K ratios strongly associated with hypertension and cardiovascular risk. Evidence from prospective cohort studies—most notably ELSA-Brasil—establishes temporally ordered associations between changes in Na/K ratio and blood pressure, while the Peruvian stepped-wedge trial provides strong causal evidence that improving this ratio through salt substitution reduces blood pressure and incident hypertension. The Na/K ratio emerges as a superior biomarker compared to either mineral independently, with important implications for surveillance and intervention design.

Understanding hypertension in Latin America requires moving beyond narrow biomedical explanations to embrace socioecological frameworks that integrate biological mechanisms with cultural practices, socioeconomic inequalities, and food system transformations. While physiological research has elucidated mechanisms linking sodium and potassium to blood pressure regulation, these biological pathways operate within contexts shaped by nutrition transition, urbanization, globalization, and persistent inequities. Genetic determinants of salt sensitivity add an additional layer of heterogeneity, with Latin American populations characterized by substantial genetic admixture that remains understudied relative to populations of European or Asian ancestry.

### 11.2 Socioecological determinants

The evidence documents clear socioeconomic gradients in sodium and potassium intake, with lower-income groups facing greater exposure to high-sodium, low-potassium diets through the confluence of lower food purchasing power, greater reliance on processed foods, and more obesogenic food environments. These exposure disparities compound with differential susceptibility (higher obesity and comorbidity burden) and differential healthcare access to produce entrenched socioeconomic gradients in hypertension outcomes. The absence of explicit, published socioecological frameworks tailored to Latin American contexts represents a critical gap; the field operates with implicit rather than specified multilevel models, limiting systematic identification of intervention leverage points.

### 11.3 Intervention effectiveness and implementation challenges

Interventional evidence is limited in volume but high in quality for the salt substitution approach. The Peruvian trial provides proof-of-concept for a scalable, equity-enhancing structural intervention. However, substantial gaps remain regarding implementation at scale: cost-effectiveness under routine programmatic conditions, safety in populations with impaired kidney function, optimal delivery models, acceptability across diverse cultural contexts, and equity impacts require empirical investigation. Food system interventions—reformulation, labeling, taxation—show promise in countries that have implemented them (Chile, Brazil, Argentina) but evidence of sustained impact is limited by short evaluation periods and methodological challenges.

### 11.4 From biomedical to socioecological understanding

Interventions targeting individual behavior change without addressing structural determinants—food availability, affordability, marketing, and broader social determinants—are unlikely to achieve sustained population-level impact or reduce disparities. Structural approaches operating on environments and default options (reformulation, salt substitution, food subsidies) may be more equitable than approaches requiring individual resources, effort, and health literacy. Equity considerations must be central rather than peripheral to hypertension prevention efforts. Interventions implemented without attention to equity may preferentially benefit advantaged groups, thereby widening disparities even as population means improve—a phenomenon termed “intervention-generated inequalities” (57).

### 11.5 Methodological priorities

Substantial methodological heterogeneity limits synthesis and comparability across studies. Harmonized protocols for exposure assessment, outcome measurement, covariate adjustment, and subgroup analyses would enhance evidence quality and enable pooled analyses. Priorities include validating spot urine methods in diverse Latin American populations, establishing standardized 24-h urine collection protocols with quality control, linking dietary intake with biomarkers to quantify food sources, and developing consensus regarding handling of antihypertensive medication in analyses.

### 11.6 Limitations of this review

As with all narrative reviews, this work has several limitations that must be acknowledged when interpreting its findings. These relate both to the primary literature we synthesized and to our review methodology.

Regarding the *search and selection process*: First, while our search covered seven databases including regional repositories (LILACS, SciELO), it was not exhaustive. The possibility of incomplete retrieval—particularly of studies in regional journals with limited international indexing or grey literature—cannot be excluded, and some relevant publications may have been missed. Second, our focus on publications from 2015–2025 means that important earlier work is not systematically represented among the primary sources reviewed. While landmark pre-2015 studies are incorporated as contextual background, this temporal restriction may have excluded some relevant historical data. Third, as source selection in narrative reviews involves authorial judgment, some degree of selection bias in favor of studies that align with or illustrate the main arguments of the review cannot be entirely ruled out. We attempted to mitigate this by including a broad range of study designs and explicitly discussing contradictory evidence where it exists.

Regarding the *primary literature*: Fourth, the evidence base is dominated by observational studies with inherent limitations for causal inference, including potential confounding, measurement error, and reverse causality; interventional evidence remains limited in volume, with the Peruvian trial being a notable exception. Fifth, the marked heterogeneity in study designs, assessment methods (e.g., 24-h urine vs. spot urine), populations, and outcome definitions across the reviewed literature prevented quantitative pooling and necessitated a qualitative synthesis. Sixth, most studies focus on isolated nutrients (sodium and potassium) rather than whole dietary patterns, limiting our understanding of how these electrolytes operate within broader nutritional contexts. Seventh, the under-representation of rural populations, indigenous communities, and several smaller Latin American countries in the evidence base limits the generalizability of our conclusions to these groups.

Finally, as a *methodological limitation of the review itself*, we did not perform a formal risk of bias assessment using a structured tool for all included studies. While we have integrated critical appraisal of key bodies of evidence into the narrative, the absence of a systematic and transparent quality appraisal introduces the potential for over- or under-estimation of the strength of the evidence.

## 12 Conclusion

Reducing hypertension burden in Latin America requires comprehensive strategies addressing sodium excess and potassium deficiency through interventions operating at multiple levels—individual, interpersonal, organizational, community, and policy. Evidence supports salt substitution with potassium-enriched alternatives, food system reforms reducing sodium in processed foods, and promotion of potassium-rich dietary patterns as cost-effective approaches with potential for population-level impact. However, translation of evidence into effective, equitable, sustainable programs faces substantial implementation challenges rooted in food system economics, industry resistance, cultural practices, socioeconomic inequalities, and health system capacity constraints.

Addressing these challenges requires political commitment, multi-sectoral coordination, community engagement, and sustained investment in surveillance, research, and program implementation. Future research priorities include: standardized exposure assessment enabling trend monitoring and policy evaluation; scaled intervention trials testing delivery models and implementation strategies in diverse settings; food-source attribution informing targeted reformulation; long-term outcome studies examining cardiovascular endpoints and effect modification; economic evaluations assessing cost-effectiveness and equity impacts; and development of socioecological frameworks guiding multilevel intervention design.

The complexity of this multifactorial problem should not paralyze action. Evidence is sufficient to justify implementation of proven interventions while simultaneously conducting research to refine approaches, address gaps, and ensure equity. As Rose (58) articulated, small shifts in population mean exposures yield large reductions in disease burden—the population prevention paradox. Modest reductions in sodium intake and improvements in sodium-potassium balance across Latin American populations would prevent thousands of cardiovascular events and deaths annually, with benefits accruing disproportionately to disadvantaged groups bearing the highest burden. Ultimately, addressing hypertension in Latin America is both a technical challenge requiring rigorous science and an ethical imperative requiring political will, structural reforms, and commitment to health equity.

## Supplementary material

Supplementary Table S1

Complete database search strings for all seven databases including filters applied.

Supplementary Figure S1

PRISMA-style flow diagram detailing the study selection process.
