# Supplementary material for: Sodium, potassium, and blood pressure regulation in Latin American populations: a critical narrative review of multifactorial determinants
Source: Front Cardiovasc Med. 2026 Apr 10;13:1770880. doi: 10.3389/fcvm.2026.1770880 (PMC13106571; doi:10.3389/fcvm.2026.1770880)
Supplement: Supplementary file 1 [file Datasheet1.pdf]

Supplementary Material: Methodological Transparency and Search Strategies

Torres A., Sanchez-Redroban J.D., Trujillo G., Perez A.

February 2026

Overview

This document contains the detailed search strategies, eligibility criteria, and the study selection process (PRISMA 2020) for the manuscript: *"Sodium, Potassium, and Blood Pressure Regulation in Latin American Populations: A Critical Narrative Review"*.

Section 1: Detailed Search Strategies

To ensure reproducibility, Table S1 lists the exact search strings used across seven electronic databases. The search was conducted for the period 2015–2025.

Table S1: Complete search strings and results per database.

Database	Search Strategy (Terms and Boolean Operators)	Hits (N)
**PubMed**	((”sodium, dietary”[MeSH] OR ”sodium chloride, dietary”[MeSH] OR ”sodium intake”[tiab] OR ”salt intake”[tiab]) AND (”potassium, dietary”[MeSH] OR ”potassium intake”[tiab]) AND (”hypertension”[MeSH] OR ”blood pressure”[MeSH]) AND (”latin america”[MeSH] OR ”brazil”[MeSH] OR ”mexico”[MeSH] OR ”ecuador”[MeSH] OR ”hispanic or latino”[MeSH]))	842
**LILACS**	(tw:((sodio OR sodium OR sal OR salt) AND (potasio OR potassium))) AND (tw:(hipertensión OR hypertension OR ”presión arterial”))	428
**SciELO**	(sodium OR sodio OR salt) AND (potassium OR potasio) AND (hypertension OR hipertensión)	315
**Scopus**	TITLE-ABS-KEY ((”sodium intake” OR ”salt intake”) AND (”potassium intake”) AND (”hypertension” OR ”blood pressure”) AND (”latin america” OR ”south america” OR ”caribbean”))	760
**Web of Science**	TS=((”sodium intake” OR ”salt intake”) AND (”potassium intake”) AND (”hypertension” OR ”blood pressure”) AND (”Latin America”))	684
**Google Scholar**	”sodium intake” AND ”potassium intake” AND hypertension AND (”Latin America” OR ”South America”) (First 200 relevant results analyzed)	1,200
**SciSpace**	(”sodium intake” OR ”salt intake”) AND (”potassium intake”) AND (”hypertension”) AND (”Latin America”)	185
**Total Records**	**Sum of all identified records**	**4,414**

Section 2: Eligibility Criteria (PICOS Framework)

The inclusion and exclusion criteria were predefined to minimize selection bias.

Table S2: Inclusion and Exclusion Criteria.

Criterion	Inclusion Criteria	Exclusion Criteria
**Population**	Adults ($\geq$19 years) residing in Latin American countries or territories.	Pediatric populations, pregnant women, or Latin American migrants living outside the region.
**Exposure**	Dietary sodium and potassium intake (measured by 24h urine, spot urine, or dietary surveys).	Studies focusing solely on other minerals (e.g., magnesium, calcium) without salt data.
**Outcomes**	Systolic/Diastolic BP levels, prevalence of hypertension, salt-sensitivity markers.	Case reports, animal models, or *in vitro* studies.
**Study Design**	Observational (cross-sectional, cohort), clinical trials, and systematic reviews.	Editorials, letters to the editor, or conference abstracts without full data.
**Language**	English, Spanish, and Portuguese.	Languages other than the three specified.

Section 3: Study Selection Process (PRISMA 2020)

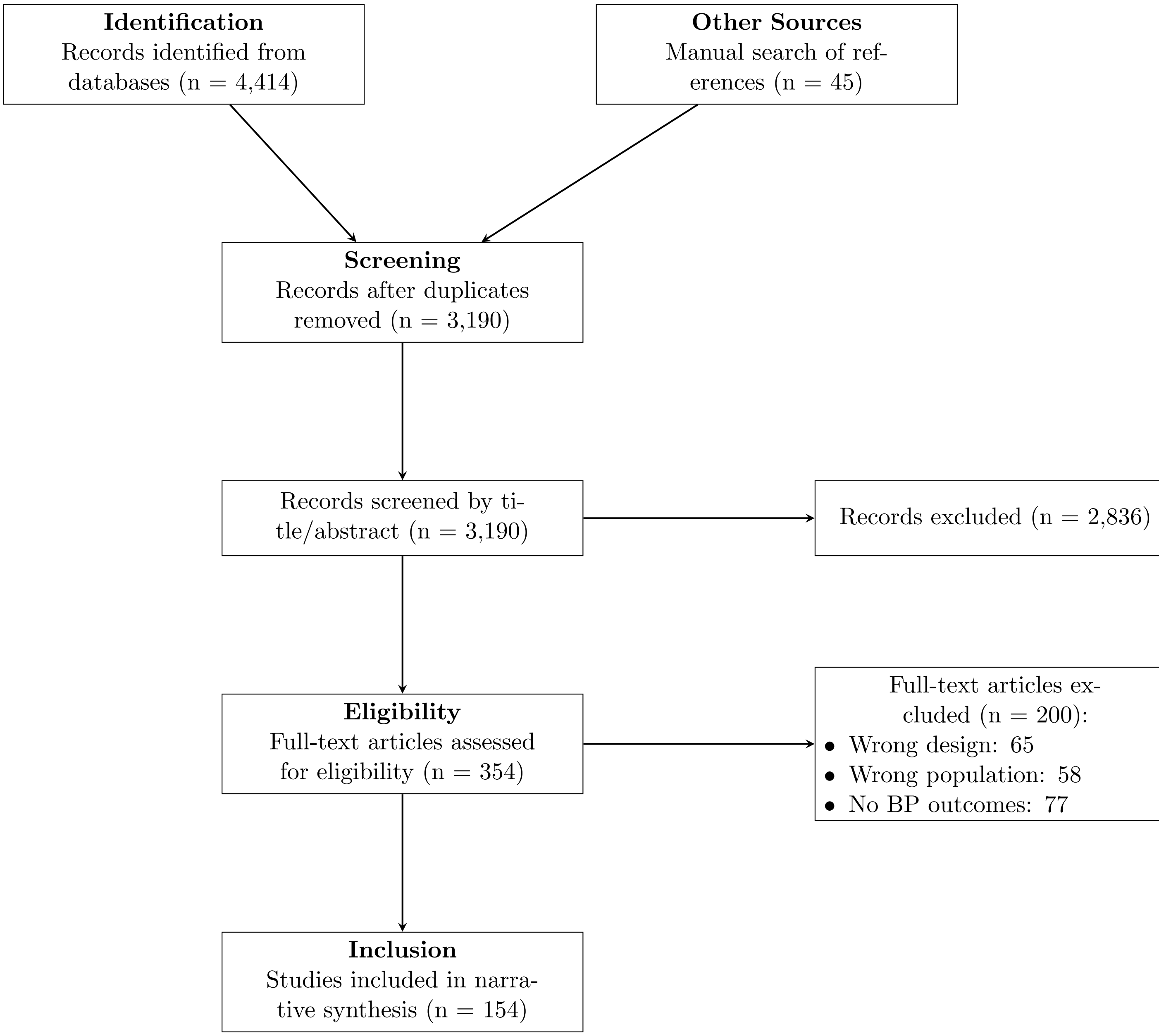

Figure S1: PRISMA 2020 flow diagram illustrating the systematic selection process.
